# 645. Expedited Partner Therapy Following Gonorrhea/Chlamydia Infection Among Sexual Minority Men in Peru: A Randomized Controlled Trial

**DOI:** 10.1093/ofid/ofaf695.209

**Published:** 2026-01-11

**Authors:** Jesse ClarkCatherine Oldenburg, Jessica Gutierrez, Rolando Valladares, Elena Domador, JoseLuis Castro, Narendar Kumar, Jose Ipanaque, Cherie Blair, Robinson Cabello

**Affiliations:** Proctor Foundation for Research in Ophthalmology, University of California San Francisco, San Francisco, California; Via Libre, Lima, Lima, Peru; Via Libre, Lima, Lima, Peru; Via Libre, Lima, Lima, Peru; Via Libre, Lima, Lima, Peru; VIa Libre, Lima, Lima, Peru; Via Libre, Lima, Lima, Peru; David Geffen School of Medicine at UCLA, Los Angeles, California; Via Libre, Lima, Lima, Peru

## Abstract

**Background:**

Expedited partner therapy (EPT) reduces the risk of recurrent Neisseria gonorrheae (GC) and Chlamydia trachomatis (CT) infection among heterosexuals but its effect among sexual minority men (SMM) remains uncertain. We tested the impact of EPT on partner notification (PN) and recurrent GC/CT infection among SMM in Lima, Peru.

**Figure:**
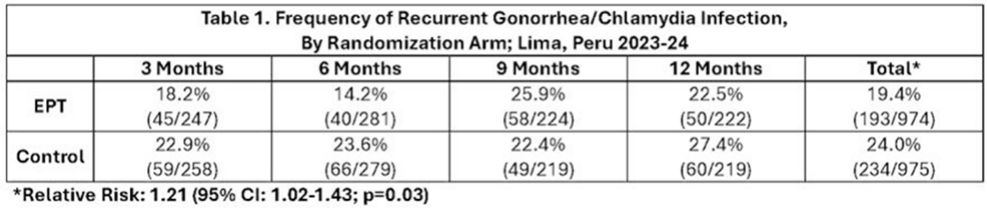

**Methods:**

We screened 2,428 SMM for GC/CT at any anatomic site between May, 2022 and May, 2023. Individuals with GC/CT were randomly assigned to receive either EPT (antibiotic treatment packets for five of their recent sexual partners) or standard-of-care counseling. Participants returned after 3-4 weeks for repeat STI testing and to report notification outcomes. Participants were followed for 12 months with quarterly HIV/STI testing and partner management according to their original randomization. The frequency of self-reported partner notification and laboratory-diagnosed recurrent GC/CT infection were compared between arms.

**Results:**

We enrolled 559 SMM with GC (36.1% rectal; 24.3% pharyngeal; 8.9% urethral) and/or CT (42.2% rectal; 5.0% pharyngeal; 9.1% urethral) infection. Median age was 29 years, with 11 partners reported over the previous three months. HIV prevalence at baseline was 50.3% and 18.8% had untreated syphilis (RPR>1:16). Among participants provided EPT, 82.2% reported notifying at least one partner of their exposure, compared to 66.7% of control arm participants (OR: 2.32, 95% CI: 1.56, 3.45), with greater numbers of participants in the EPT arm notifying at least one of their primary (77.0% vs. 67.1%; OR: 1.64 [0.81, 3.37] or casual (58.3% vs. 42.8%; OR: 1.87 [1.34, 2.63] partners. The overall notification frequency was similar between the EPT and Control arms (8.7% and 8.2% of all partners notified, respectively). The cumulative frequency of recurrent GC/CT infection over the 12-month follow-up period was 19.8% (193/974) in the EPT arm and 24.0% (234/975) in the Control arm (Relative Risk: 1.21, 95% CI: 1.02-1.43).

**Conclusion:**

Expedited partner therapy improves partner notification and reduces the risk of recurrent infection among SMM with GC/CT. Additional research is needed to determine the public health impact of EPT on HIV and STI transmission and to optimize intervention delivery.

**Disclosures:**

All Authors: No reported disclosures

